# Percutaneous treatment of vascular access-site complications: a ten years’ experience in two centres

**DOI:** 10.1186/s42155-020-00120-7

**Published:** 2020-06-08

**Authors:** Roberto Minici, Sara Paone, Marisa Talarico, Lorenzo Zappia, Karim Abdalla, Maria Petullà, Domenico Laganà

**Affiliations:** 1 Radiology Division, Department of Experimental and Clinical Medicine, Magna Graecia University of Catanzaro, University Hospital Mater Domini, Viale Europa, 88100 Catanzaro, CZ Italy; 2 IRC - FSH, Department of Health Sciences, Magna Graecia University of Catanzaro, Catanzaro, Italy; 3 Cardiology Division, Department of Biomedical, Metabolic and Neural Sciences, University of Modena and Reggio Emilia, Policlinico di Modena, Modena, Italy; 4 Anaesthesia and Intensive Care Unit, Department of Medical and Surgical Sciences, Magna Graecia University of Catanzaro, University Hospital Mater Domini, Catanzaro, Italy; 5 Radiology Division, University of Insubria, Varese, Italy

**Keywords:** Pseudoaneurysm, Retroperitoneal bleedings, Dissections, Percutaneous treatment, Vascular access-site complications

## Abstract

**Background:**

The spread of percutaneous arterial catheterization in diagnostic and therapeutic procedures has led to a parallel increase of vascular access site complications. The incidence of these events is between 0.2–1%. A detailed analysis of injuries by type of procedure shows a higher incidence of injuries after therapeutic procedures (3%) than those observed for diagnostic ones (1%), due to the greater size of the vascular devices used and the necessity to frequently administer anticoagulant and antiplatelet therapy during procedures. The iatrogenic arterial injuries requiring treatment are the pseudoaneurysm, arteriovenous fistula, arterial rupture and dissection. Less frequent complications include distal embolization of the limbs, nerve damage, abscess and lymphocele.

Moreover, the use of percutaneous vascular closure devices (VCD) has further expanded the types of complications, with an increased risk of stenosis, thrombosis, distal embolism and infection. Our work aims to bring the personal 10 years’ experience in the percutaneous treatment of vascular access-site complications.

**Results:**

Ninety-two pseudoaneurysms (PSA), 12 arteriovenous fistulas (AVF), 15 retrograde dissections (RD) and 11 retroperitoneal bleedings (RB) have been selected and treated. In 120/130 cases there were no periprocedural complications with immediate technical success (92.3%). Nine femoral PSA, treated with percutaneous ultrasound-guided thrombin injection, showed a failure to close the sac and therefore they were treated by PTA balloon inflation with a contralateral approach and cross-over technique. Only one case of brachial dissection, in which the prolonged inflation of the balloon has not led to a full reimbursement of the dissection flap, was then surgically repaired. At the 7 days follow-up, complications were two abscesses in retroperitoneal bleedings, treated by percutaneous drainage. At 3 months, acute occlusion of 3 covered femoral stents occurred, then treated by loco-regional thrombolysis and PTA. A total of 18 major complications was recorded at 2 years, with a complication rate at 2 years of 13.8%.

**Conclusions:**

The percutaneous treatment of vascular access-site complications is the first-choice treatment. It represents a safe and effective option, validated by a high technical success rate and a low long-term complication rate, that allows avoiding the surgical approach in most cases.

## Background

The spread of percutaneous arterial catheterization in diagnostic and therapeutic procedures has led to a parallel increase of vascular complications at the access site. The incidence of these events is between 0.2–1% (Tsetis 2010).

A detailed analysis of injuries by type of procedure (diagnostic versus interventional) shows a higher incidence of injury after therapeutic procedures (3%) than observed for diagnostic procedures (1%), due to the greater size of the vascular devices used and the necessity to frequently administer anticoagulant and antiplatelet therapy during procedures (Tonnessen 2011; Keeling et al. 2009; Katzenschlager et al. 1995).

Concerning puncture site, in literature is described a prevailing incidence of complications at the femoral access (Katzenschlager et al. 1995), as the common femoral artery (CFA) is by far the most common access site for endovascular procedures. The complications of radial and popliteal access sites are less frequent, while brachial and axillary access sites are rarely used in comparison to the femoral and radial ones (Romaguera et al. 2012; Xiong et al. 2012; Johnson et al. 1994; Reich et al. 2017; Ortiz et al. 2014).

The iatrogenic arterial injuries requiring treatment are pseudoaneurysm, arteriovenous fistula, arterial rupture and dissection. Less frequent complications include distal embolization of the limbs, nerve damage, abscess and lymphocele (Tsetis 2010; Tonnessen 2011; Keeling et al. 2009; Katzenschlager et al. 1995; Romaguera et al. 2012; Xiong et al. 2012; Johnson et al. 1994). Moreover, the use of percutaneous vascular closure devices (VCD) has further expanded the types of complications, with an increased risk of stenosis, thrombosis, distal embolism and infection (Cianci et al. 2013).

The success of surgical repair of vascular complications is close to 100%, but such treatments are associated with a rate of post-operative morbidity up to 25%, with a mortality rate up to 3.5%, because of the significant comorbidities (Morgan and Belli 2003; Thalhammer et al. 2000).

Percutaneous treatment is a valid alternative to surgery. These procedures are performed under local anaesthesia and are usually well-tolerated, associated with lower costs and shorter hospitalization compared to surgery.

Our work aims to bring the personal experience of 10 years in the multimodal treatment of the complications of the percutaneous vascular access site.

## Methods

### Study design and population

The Institutional Review Board approval and informed written consent from each patient have been obtained. This study is a two-centers (Insubria University of Varese and Magna Graecia University of Catanzaro, Italy), retrospective analysis of prospectively collected data of consecutive patients undergone percutaneous treatment of vascular access-site complications, from September 2009 to September 2019. Inclusion criteria are: I) major vascular access site complications: pseudoaneurysms (PSA), arteriovenous fistulas (AVF), retrograde dissections (RD) and retroperitoneal bleedings (RB); II) evaluation by a multidisciplinary team of vascular surgeons, interventional radiologists and anaesthetists; III) refusal of surgical approach by patients or being considered unfit for surgery when surgery has been considered the better choice among treatment options by the multidisciplinary team. The main exclusion criteria is a glomerular filtration rate (GFR) < 30 mL/min in non-dialyzing patients, requiring i.v. administration of iodine contrast media.

The clinical diagnosis was confirmed by Doppler Ultrasound (DUS) (Acuson Sequoia 512; Philips iU22) and/or CT angiography (Light-SpeedPlus®, GE, Milwaukee, USA; Angio-CT Aquilion 64, Toshiba, Tokyo, Japan).

In the case of PSA, the size of the aneurismal sac, the vessel of origin and the size of the neck (narrow/wide) were evaluated by Doppler ultrasound; PSA were classified as simple (unilocular) and multilocular.

In the case of AVF, A-V communication, as well as the velocimetry gradient through the AVF, were documented by the assessment of peak systolic velocity, which allowed us to classify them into low and high flow AVF. Doppler ultrasound was integrated with CT-angiography, in patients presenting retroperitoneal bleeding.

The RD of the iliac axis, initially diagnosed with Doppler ultrasound, were also submitted to CT-angiography, to assess accurately the extent of the dissection and the presence of thrombosis and/or occluding flap.

### Description of treatments

**Fig. 1:**
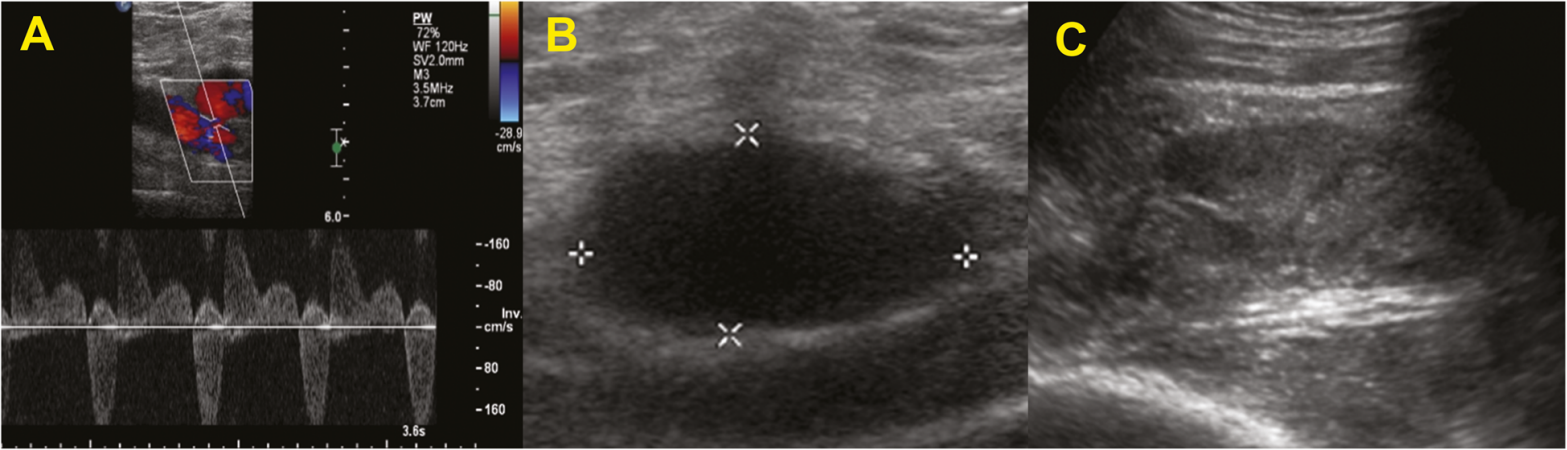
**a-c** Pseudoaneurysm (PSA) embolized with thrombin. Doppler US view (**a**) of a common femoral artery PSA with a small neck. Pre-treatment PSA, B-mode examination (**b**). Thrombosed PSA (**c**) after treatment with thrombin injection under ultrasound guidance

The type of treatment of femoral PSA was mainly dictated by the morphology (uni/multiloculated), and by the size of the neck. According to the sonographic appearance, the femoral unilocular PSA with small neck were treated by percutaneous approach with ultrasound guidance using a 15–7 MHz probe (Philips iU22), under sterile conditions, with the injection of bovine thrombin (1000 U/ml, D-STAT, Flowable Hemostat, Vascular Solution, NGC Medical Italy), injected into the aneurismal sac with a 22 gauge needle (Fig. 1). The amount of thrombin solution was determined by direct vision of the sac with doppler ultrasound (average values of length of the neck < 1 cm and dimensions from 3 to 6 cm), to assess the formation of a clot until the complete filling of the lumen and the disappearance of the Color flow Doppler signals. The unilocular PSA with wide neck were treated by blocking flow with cross-over approach, with contralateral access, placing a catheter for transluminal angioplasty (PTA) with a balloon dilator of the calibre of 6–8 mm and a length of 4 cm (Cordis, Warren, NJ, USA); the balloon was dilated near the neck of the PSA, for about 10 to 15 min and, once the flow within PSA was excluded, thrombin was injected under ultrasound guidance.

**Fig. 2:**
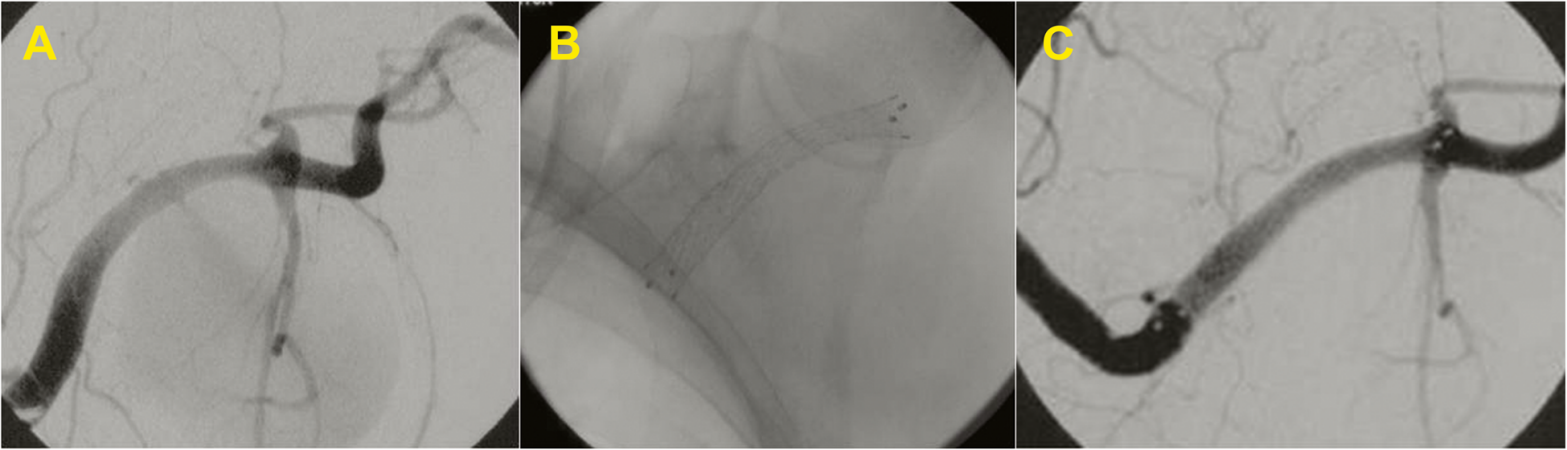
**a-c** Pseudoaneurysm (PSA) treated with a stent-graft. Massive iatrogenic axillary artery PSA: **a** angiography shows the small tract through the arterial wall which feeds the PSA. **b** Placement of a covered stent along the axillary artery; **c** control angiogram after procedure shows complete exclusion of the PSA with regular patency of subscapular artery

The remaining multilocular femoral PSA were treated with Nitinol PTFE-covered stent-graft (Fluency - Bard, Tempe, AZ, USA) (7/8/9 mm of diameter and length of 3–4 cm). A femoral angiography from an ipsilateral oblique view (e.g., the right anterior oblique [RAO] for right femoral artery) was performed to best displays the bifurcation of the profunda and superficial femoral branches. Hence, a femoral angiography from an oblique projection indicates the suitability of the device insertion, allowing to check the presence of an adequate landing zone in the CFA respect to the origin of the profunda femoral artery. The PSA localized in axillary artery were treated with Nitinol PTFE-covered stent-graft (Fluency - Bard, Tempe, AZ, USA) by placing a 60 cm, 5–6 Fr long introducer into the subclavian artery to assess the exact site of the lesion; it was later advanced a stent coated on a short 7 cm introducer, 9 Fr calibre, via the transfemoral route (Fig. 2). The remaining PSA located in BA were all treated by percutaneous injection of thrombin under ultrasound guidance.

The high-flux symptomatic AVF were unfit for surgery, due to cardiovascular comorbidities, so they were treated endovascularly by placing microcoils along the AV fistula or with Nitinol PTFE-covered stent-graft (Fluency - Bard, Tempe, AZ, USA).

The RB were treated by embolization with microcoils or, in cases of iliac arteries involvement, with Nitinol PTFE-covered stent-graft (Fluency - Bard, Tempe, AZ, USA). It should be pointed out that the diagnosis of rupture of the iliac artery occurred during a cardiologic procedure and the treatment was carried out by a team of radiologists in the interventional cardiology room.

**Fig. 3:**
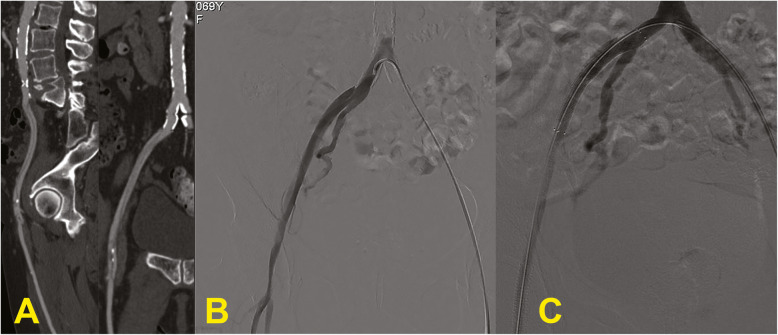
**a-c** Dissection. Sagittal and coronal CT reformats (**a**) show dissection of the whole iliac axis extending up to the common femoral artery. Angiography with crossover approach (**b**) confirms the dissection with iliac stenosis proximally to the femoral bifurcation. The dissection has been treated with the placement of two stents; angiography after treatment (**c**) demonstrates restoration of regular patency of iliac and common femoral arteries

The RD, involving iliac or brachial artery, were treated, respectively, by Nitinol coated stent SMART type (Cordis, Johnson & Johnson, Miami Lakes, FL, USA) with cross-over technique (Fig. 3) and by prolonged balloon inflation, to facilitate the intimal reimbursement. There is a scarcity of data in the literature regarding the optimal inflation time to achieve the best outcome with balloon angioplasty treatment of retrograde arterial dissections. However, the optimal inflation time is likely similar to that recorded to minimize dissections after balloon angioplasty treatment of peripheral arterial disease, so a more than 3 min of inflation time (Horie et al. 2018). Although few, the cases reported in this study anecdotally confirm this finding.

### Follow-up

Except for RB, which were clinically monitored, all patients who had immediate technical success, were subjected to clinical and radiological follow-up until discharge. It has been considered as a major complication, a condition needing a percutaneous/surgical treatment after the first percutaneous treatment had been performed. Instrumental checks were performed by Doppler ultrasound at 1,3,6 and 12 months; subsequently, the 24 covered stents (12 femoral PSA, 3 axillary PSA, 7 AVF and 2 retroperitoneal hematomas of the iliac artery) were subjected to annual Doppler ultrasound exams.

### Statistical analysis

Data were maintained in an Excel spreadsheet (Microsoft Inc., Redmond, Wash) and the statistical analyses were performed on an intention-to-treat basis, using SPSS software (SPSS Inc., Chicago IL). Kolmogorov-Smirnov test and Shapiro-Wilk test were used to verify the normality assumption of data. Categorical data are presented as frequency (percentage value). Continuous normally distributed data are presented as mean ± standard deviation. Continuous not normally distributed data are presented as median (interquartile range).

## Results

In the period between September 2009 and September 2019, 130 patients (84 males, 46 females), aged between 40 and 92 years (66.6 ± 12.8 years), were treated (Table 1). We selected and treated 92 pseudoaneurysms (PSA), 12 arteriovenous fistulas (AVF), 15 retrograde dissections (RD) and 11 retroperitoneal bleedings (RB). It should be emphasized that popliteal accesses have never been used in our experience.

**Table 1:** Population data

Variables		All patients (***n*** = 130)
Age (years), mean (± SD)		66.6 (±12.8)
Male, n (%)		84 (64%)
Vascular access-site complications, n (%)		130 (100%)
*Pseudoaneurysms, n (%)*		*92 (71%)*
	Common femoral artery	57 (44%)
	Superficial femoral artery	22 (17%)
	Deep femoral artery	5 (4%)
	Brachial artery	5 (4%)
	Axillary artery	3 (2%)
*Arteriovenous fistulas, n (%)*		*12 (9%)*
	CFA - CFV	2 (1.5%)
	SFA - CFV	7 (5%)
	DFA - CFV	3 (2.5%)
*Retroperitoneal bleedings, n (%)*		*11 (9%)*
	Superficial epigastric artery	5 (4%)
	Circumflex iliac artery	3 (2.5%)
	Lumbar artery	1 (1%)
	External iliac artery	2 (1.5%)
*Retrograde dissections, n (%)*		*15 (11%)*
	Brachial artery	3 (2.5%)
	External iliac artery	12 (8.5%)

PSA were localized mainly at the level of the femoral artery (84 out of 92): 57 in the common femoral artery (CFA), 22 in the superficial femoral artery (SFA) and the remaining 5 in the deep femoral artery (DFA). In the remaining 8 cases, PSAs were located in the upper limbs, 3/8 in the axillary artery (AA), treated by placement of Nitinol PTFE-covered stent-graft, and 5/8 in the brachial artery (BA), treated by ultrasound-guided injection of bovine thrombin. PSA located at the level of femoral artery presented a unilocular small neck in 64 cases, treated by ultrasound-guided injection of bovine thrombin, a unilocular wide neck in 8 cases, treated by blocking flow placing a balloon for transluminal angioplasty (PTA), and a multilocular neck in 12 cases, treated by placement of Nitinol PTFE-covered stent-graft.

**Fig. 4:**
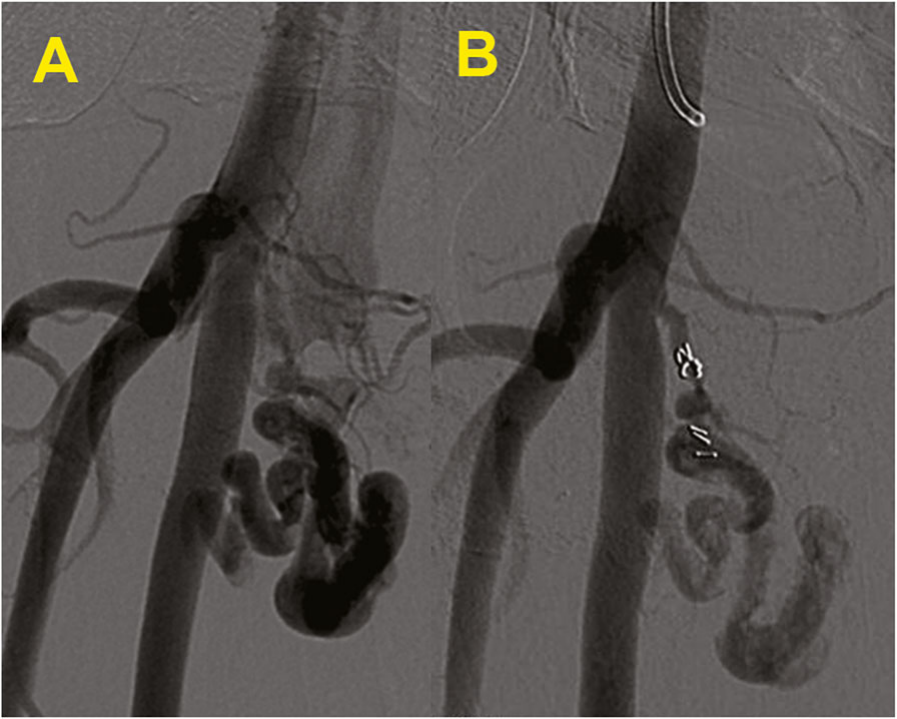
**a-b** Arteriovenous fistula (AVF). Angiography (**a**) demonstrate a small fistula tract between the origin of the superficial femoral artery and vein; the tract has been embolized with microcoils with a significant reduction of blood flow through the fistula at the angiography performed immediately after the embolization (**b**)

The AVF, all of them of high-flux type, were located in 2 cases between the CFA and the common femoral vein (CFV), in 7 cases between the SFA and the CFV, and the remaining 3 cases between the DFA and the CFV. They were treated endovascularly by placing microcoils along the AV fistula (5 cases out of 12; Fig. 4); in the remaining cases (7/12), the treatment was performed with Nitinol PTFE-covered stent-graft (Fluency - Bard, Tempe, AZ, USA).

The RB were in the number of 11, caused by lesions of 5 superficial epigastric arteries, 3 circumflex iliac arteries, and 1 lumbar artery, treated by embolization with microcoils, and by lesions of 2 external iliac arteries, treated with Nitinol PTFE-covered stent-graft (Fluency - Bard, Tempe, AZ, USA).

The 15 RD were located in 3 cases at the level of BA, treated by prolonged balloon inflation, and in 12 cases at the level of the external iliac artery - 2 of which were associated with occluding thrombosis of the dissection flap – treated by placement of Nitinol-coated stent SMART-type (Cordis, Johnson & Johnson, Miami Lakes, FL, USA) with cross-over technique.

The treatments performed are shown in detail in Table 2.

**Table 2:** Treatments

Vascular access-site complications	Treatments	
*Pseudoaneurysms, n*		*92*
	Ultrasound-guided injection of bovine thrombin, n (%)	69 (75%)
	Stenting, n (%)	15 (16%)
	PTA balloon inflation, n (%)	8 (9%)
*Arteriovenous fistulas, n*		*12*
	Stenting, n (%)	7 (58%)
	Microcoils, n (%)	5 (42%)
*Retroperitoneal bleedings, n*		*11*
	Microcoils, n (%)	9 (81%)
	Stenting, n (%)	2 (19%)
*Retrograde dissections, n*		*15*
	Stenting, n (%)	12 (80%)
	PTA balloon inflation, n (%)	3 (20%)
*All, n (%)*		*130 (100%)*
	Ultrasound-guided injection of bovine thrombin, n (%)	69 (53%)
	Stenting, n (%)	36 (28%)
	Microcoils, n (%)	14 (11%)
	PTA balloon inflation, n (%)	11 (8%)

In 120/130 cases an immediate technical success was reached (92.3%). Nine femoral PSA, treated with percutaneous ultrasound-guided thrombin injection, showed a failure to close the sac and therefore they were treated by PTA balloon inflation with a contralateral approach and cross-over technique. Only one case of brachial dissection, in which the prolonged inflation of the balloon has not led to a full reimbursement of the dissection flap, was then surgically repaired. At 7 days, 2 retroperitoneal bleedings became abscessualized, treated with a 12 Fr percutaneous drainage and oral antibiotics, with resolution in 7–10 days. At 3 months, the acute occlusion of 3 of the 24 covered stents placed in the superficial femoral artery was observed during Doppler ultrasound control; these were successfully treated with loco-regional thrombolysis and PTA. Doppler ultrasound performed at 6 months and 12 months demonstrate myointimal hyperplasia inside the stent in all cases without significant stenosis. At 2 years follow-up, 3 symptomatic patients, due to significant stenosis of the proximal or distal edge of the stent-graft (2 in the SFA and 1 in the axillary artery), have been successfully retreated with PTA alone. Considering the major complications as stated above, a total of 18 major complications was recorded at 2 years, with a complication rate at 2 years of 13.8%, as shown in detail in Table 3.

**Table 3:** Major complications

Follow-up	New major Complications		Complication rate
Periprocedural, n (%)		10 (55%)	7.7%
	Persistent pseudoaneurysm, n	9	
	Persistent dissection, n	1	
At 7 days, n (%)		2 (11%)	9.2%
	Abscess, n	2	
At 3 months, n (%)		3 (17%)	11.5%
	Stent thrombosis, n	3	
At 2 years, n (%)		3 (17%)	13.8%
	Stent thrombosis, n	3	
All, n (%)		18 (100%)	

## Discussion

The number of percutaneous endovascular procedures has grown rapidly thanks to the technological advances of materials, long term satisfactory clinical results and lower morbidity when compared to traditional surgery. Although the number and the complexity of the procedures are rising, the incidence of local complications has not substantially changed over the years (Tsetis 2010; Kopin et al. 2019).

The arteries of the upper limb (radial, brachial, and rarely axillary) are used as arterial access, especially in cardiologic procedures. The trans-radial approach is associated with a lower incidence of major complications when compared to femoral approach (Rigattieri et al. 2016); the most common major complication is the occlusion of the radial artery, which rarely leads to clinical manifestations, due to the dual arterial supply guaranteed at the hand (Reich et al. 2017). The major complications associated with the brachial approach are thrombosis, PSA and nerve compression; ischemic complications (dissection/thrombosis) are more common at this level than in femoral approach (Romaguera et al. 2012; Johnson et al. 1994).

At the level of lower limbs, the popliteal access is the least common (sometimes used in the recanalization of chronic occlusions of the femoropopliteal arterial axis) (Ortiz et al. 2014); major complications are dissection and PSA (Chan and Common 2004).

The CFA is by far the most used vascular access because it has several advantages over other sites: the large calibre, which allows the introduction of larger catheters and easier cannulation; the femoral head underneath guarantee haemostasis with manual compression (Tsetis 2010; Tonnessen 2011; Keeling et al. 2009; Reich et al. 2017). Due to the highest number of procedures performed via this route, femoral access is subject to more frequent complications than other arterial access (Ortiz et al. 2014).

Bleeding is the most frequent complication; hematoma may present as stable or unstable (uncontrolled bleeding), retroperitoneal haemorrhage or PSA. The incidence of femoral PSA varies from 0.1% to 1.5% after diagnostic angiography and up to 7.7% after interventional procedures and increases with the complexity of procedures, patient age and the presence of bleeding disorders (Graham et al. 1992; Carey et al. 2001; Erol et al. 2015).

SFA or DFA accesses, compared with CFA accesses, are more likely to lead to PSA or AVF formation, due to the smaller size and the lack of bone support against which compress after sheath removal (Morgan and Belli 2003).

The natural history of iatrogenic femoral PSA is uncertain (Graham et al. 1992). In a case series of 147 patients, Thalhammer et al. (2000) reported that 86% had a spontaneous resolution (PSA and AVF) after a mean of 23 days. Although the rupture of the femoral PSA can be a life-threatening condition (Graham et al. 1992), some authors believe sufficient Doppler ultrasound observation alone, especially for asymptomatic patients not receiving anticoagulation therapy with little PSA (diameter ≤ 3 cm) (Johns et al. 1991).

Surgical exploration of a pseudoaneurysm is often challenged by the presence of a haematoma. After the evacuation of the haematoma, another difficulty is the identification of the bleeding site, that may be more than one. Finally, surgical treatment may require simple suture of the defect or a patch angioplasty (surgical repair using a patch). Considering the aforementioned limitations, surgical repair is indicated in the cases of haemodynamically relevant bleeding or shock with a rapidly expanding haematoma, risk of skin necrosis due to pressure by the haematoma and infectious pseudoaneurysm to ensure debridement of infected tissue (Tisi and Callam 2006; Savolainen et al. 2011). In 1991, the ultrasound-guided compression was suggested (Fellmeth et al. 1991); the advantages are its simplicity, low cost and lack of ionizing radiation. However, this procedure has some limitations, related to the pain threshold of the patient and the time necessary to obtain complete closure. Moreover, failure is increased in patients on anticoagulant therapy (Hajarizadeh et al. 1995).

In 1997, Liau et al. (1997) reported the successful use of percutaneous injection of thrombin with ultrasound guidance for the closure of 5 cases of PSA of the CFA. In the series of 15 patients reported by Brophy et al. (2000), all the PSA have been successfully treated with 500–1000 U of bovine thrombin, regardless of the PSA size.

Later, in a series of 54 PSA (divided into simple, 45, and complex, 9) reported by Sheiman et al. (2001), the technical success was 50/54, with the possibility of a second approach in case of failure. In some cases (2–4%) you can proceed to a further ultrasound-guided injection procedure with an overall high success rate. However, in literature, the ultrasound-guided injection technique has a failure rate of 3% -14% (Maleux et al. 2003).

The greatest risk from thrombin injection is distal embolization (which can cause serious complications up to limb loss). Pezzullo et al. (2000) have described distal embolization in one of the 23 patients studied. The placement of the tip of the needle at distance from the neck of the pseudoaneurysm under ultrasound guidance and the slow injection of the drug under ultrasound control minimizes the risk (Maleux et al. 2003; Pezzullo et al. 2000).

Other side effects of thrombin injection include hypotension and bradycardia, bleeding - because of an ‘acquired inhibition of coagulation factor (XI) secondary to immune cross-reactivity of bovine thrombin - and anaphylactic reactions in patients who have had repeated exposure to bovine thrombin (Pope and Johnston 2000). Different forms of thrombin are commercially available, the majority of which are of bovine origin and have been used for many years (Pezzullo et al. 2000; Samal et al. 2001). Because of these risks is currently preferred the human thrombin, which implies a slightly higher cost (Vázquez et al. 2005).

The use of human thrombin would not entail the risks associated with bovine thrombin or any other immunological risk; it must be acknowledged, however, that there may be a small risk of infection, although not yet confirmed (Vázquez et al. 2005).

Quarmby et al. (2002) described the embolization of 10 PSA through the use of autologous thrombin with immediate technical success in 7 cases and the use of the new administration of the same in 3 cases.

Loose and Haslam described the percutaneous technique of blocking flow through percutaneous balloon angioplasty (PTA) inflated for about 15 min to prevent distal embolization during injection under ultrasound guidance in the sac of the PSA. Their method was effective (12/13 cases successfully treated without complications), but is rather expensive and employ ionizing radiation (Loose and Haslam 1998).

The use of stent-graft in the treatment of aortoiliac disease has recently led several authors to employ these devices to exclude aneurysms and peripheral PSA (Xiao et al. 2012; Laganà et al. 2002). In a larger series of stent-graft used in the treatment of pseudoaneurysms and AVF, Waigand et al. (1999) and Thalhammer et al. (2000) reported a technical success rate of 84–88%. However, there are limits to the indication, as the stent-graft may result in the occlusion of the DFA; theoretically, this complication can prevent the use of the site as future access. Hence, a femoral angiography from an oblique projection should be performed to evaluate the suitability of the device insertion, as it allows to check the presence of an adequate landing zone in the CFA respect to the origin of the profunda femoral artery. Besides, hip stress can lead to an increased risk of stent thrombosis (Thalhammer et al. 2000; Xiao et al. 2012).

Retroperitoneal bleedings (RB) are potentially fatal and not easy to diagnose in clinically haemodynamically stable patients; RB may occur spontaneously in the presence of coagulopathy, use of combined antiplatelet drugs (eg. dual antiplatelet therapy), oral anticoagulant therapy with heparin or poorly controlled partial thromboplastin. Generally, the target population includes elderly population. Usually, retroperitoneal hematomas present with abdominal and back pain, less frequently with fatigue, nausea, headache, and dyspnea (Baekgaard et al. 2019; Dolapsakis et al. 2019).

Besides, the RB may be iatrogenic; in particular, they may occur as a complication of arterial or venous catheterization during endovascular procedures. The retroperitoneal bleedings may result from the femoral puncture and are spread either through the Retzius space or directly on the muscles of the pelvic floor without the direct involvement of this space (Terotola et al. 1991).

Usually, AVF are between the SFA, the DFA and the adjacent lateral circumflex femoral vein. The majority of studies reported a high probability of spontaneous closure (Toursarkissian et al. 1997), but Kresowik et al. (1991) reported opposite results. Toursarkissian et al. (1997) reported that 86% of the non-symptomatic low-flow AVF resolves spontaneously, requiring only observation and close Doppler ultrasound monitoring every 2 weeks until 12 weeks. Until recently, the AVF symptomatic were treated surgically (Kresowik et al. 1991; Kelm et al. 2002). More recently, percutaneous treatment with stent-graft showed constant technical success, but with a significant risk of stent occlusion - medium and long-term graft (12% -17%) (Laganà et al. 2002).

Dissection is a frequent but often underdiagnosed complication of arterial catheterization because many cases are asymptomatic (Benjamin et al. 2015). During vascular access, coiling of the guidewire under fluoroscopy or resistance to passage may indicate subintimal passage with resulting retrograde arterial dissection. This complication is more common in the iliac arteries due to the presence of plaques by steno-occlusive atherosclerotic disease and to the tortuosity of these vessels. The operator should immediately recognize the dissection and evaluate the hemodynamic consequences. Generally, the flap of dissection doesn’t cause stenosis and the simple removal of the catheter and guide usually allows a spontaneous resolution, also due to the anterograde flow of blood. If the flap dissection is not felt by the sensitivity of the operator and extends for a longer distance gaining the true lumen, the flap may become occlusive - then counter - with acute ischemia, so that it is necessary an immediate repair by angioplasty or stenting (Tsetis and Belli 2004).

Despite the wide use of vascular closing devices, there are no studies that prove with certainty their superiority in terms of safety compared to manual compression. Only the study of Tavris et al. (2004) demonstrated that vascular complications are lower with the use of VCD. One hundred sixty-six thousand six hundred eighty patients of the National Registry of the American College of Cardiology database, undergoing cardiac catheterization in 2001, have been evaluated; 53,655 were treated with VCD for haemostasis, while in 113,025 was performed the manual compression. The risk of vascular complication was 1.1% in the group with VCD and 1.7% in the group with manual compression, with *p* < 0.001, statistically significant.

The evolution of metal alloys with flexible and elastic materials and the technological innovation on the porosity of the cover have now led to the use of stents with a higher patency rate and a lower rate of acute thrombosis. All this has led to greater use of devices close to the articular femoral site and the possibility of re-using the access through the stent struts, clearly with catheters and introducers of small arms. A high technical success rate (92.3%) has been achieved, with a low (13.8%) complication rate at 2 years.

Limitations of the study are the lack of long term follow up, except for the cases of stent-graft placement, the retrospectivity of the analysis and the scarcity of data in the literature, necessary to evaluate the congruence and the consistency of the data presented.

## Conclusions

The percutaneous treatment of vascular access-site complications is the first-choice treatment. It represents a safe and effective option, validated by a high technical success rate and a low long-term complication rate, that allows avoiding the surgical approach in most cases.

## Data Availability

The datasets used and/or analysed during the current study are available from the corresponding author on reasonable request.

## Abbreviations

CFA: Common femoral artery
VCD: Vascular closure devices
PSA: Pseudoaneurysms
AVF: Arteriovenous fistulas
RD: Retrograde dissections
RB: Retroperitoneal bleedings
SFA: Superficial femoral artery
DFA: Deep femoral artery
AA: Axillary artery
BA: Brachial artery
CFV: Common femoral vein
PTA: Percutaneous transluminal angioplasty
